# H1-antihistamine use and head and neck cancer risk in type 2 diabetes mellitus

**DOI:** 10.32604/or.2022.028449

**Published:** 2023-03-01

**Authors:** YI-NONG CHEN, YING-LIN CHEN, WAN-MING CHEN, MINGCHIH CHEN, BEN-CHANG SHIA, JENQ-YUH KO, SZU-YUAN WU

**Affiliations:** 1 Department of Otorhinolaryngology, Lo-Hsu Medical Foundation, Lotung Poh-Ai Hospital, Yilan, Taiwan; 2 Graduate Institute of Business Administration, College of Management, Fu Jen Catholic University, Taipei, Taiwan; 3 Artificial Intelligence Development Center, Fu Jen Catholic University, Taipei, Taiwan; 4 Department of Otolaryngology, National Taiwan University Hospital and National Taiwan University College of Medicine, Taipei, Taiwan; 5 Department of Food Nutrition and Health Biotechnology, College of Medical and Health Science, Asia University, Taichung, Taiwan; 6 Division of Radiation Oncology, Lo-Hsu Medical Foundation, Lotung Poh-Ai Hospital, Yilan, Taiwan; 7 Big Data Center, Lo-Hsu Medical Foundation, Lotung Poh-Ai Hospital, Yilan, Taiwan; 8 Department of Healthcare Administration, College of Medical and Health Science, Asia University, Taichung, Taiwan; 9 Cancer Center, Lo-Hsu Medical Foundation, Lotung Poh-Ai Hospital, Yilan, Taiwan; 10 Centers for Regional Anesthesia and Pain Medicine, Taipei Municipal Wan Fang Hospital, Taipei Medical University, Taipei, Taiwan; 11 Department of Management, College of Management, Fo Guang University, Yilan, Taiwan

**Keywords:** Type 2 diabetes, H1-Antihistamine, Head and neck cancer, Incidence, Incidence rate

## Abstract

This study aimed to examine the association between the use of H1-antihistamines (AHs) and head and neck cancer (HNC) risk in patients with type 2 diabetes mellitus (T2DM). Data from the National Health Insurance Research Database of Taiwan were analyzed for the period from 2008 to 2018. A propensity-score-matched cohort of 54,384 patients each in the AH user and nonuser groups was created and analyzed using Kaplan-Meier method and Cox proportional hazards regression. The results showed that the risk of HNC was significantly lower in AH users (adjusted hazard ratio: 0.55, 95% CI: 0.48 to 0.64) and the incidence rate was also lower (5.16 *vs*. 8.10 per 100,000 person-years). The lower HNC incidence rate in AH users (95% CI: 0.63; 0.55 to 0.73) suggests that AH use may reduce the risk of HNC in T2DM patients.

## Introduction

In 2020, head and neck cancer (HNC) was a significant global health issue, ranking as the seventh most common cancer and causing the seventh highest number of cancer deaths. The estimated number of new HNC cases in 2020 was 932,000, with 467,000 new HNC deaths [1]. The incidence and mortality of HNC continue to rise, and both are predicted to increase by approximately 25% by 2030 [1]. In 2020, head and neck cancer (HNC) was a prevalent concern in Taiwan, ranking as the third most common cancer among men and the fifth leading cause of cancer death [2–5]. HNC mostly occurs in economically active adult men; the median ages at diagnosis and death are 57 and 60 years, respectively, for men in Taiwan [2–5]. Consequently, HNC places a tremendous burden not only on public finances [6] but also on family finances because those diagnosed with HNC tend to be key providers for their families [2–4].

An estimated 537 million adults worldwide have diabetes mellitus (DM)—more than 1 in 10 adults [7]. The incidence and prevalence of DM are still increasing, with 783 million people globally predicted to be affected in 2045 [7]. Type 2 diabetes mellitus (T2DM) is the most prevalent form of diabetes, affecting hundreds of millions of people worldwide and accounting for over 90% of all diabetes cases [8]. T2DM is marked by hyperglycemia, insulin resistance, decreased insulin secretion, and dyslipidemia, including elevated triglyceride levels and low high-density lipoprotein cholesterol [9–12]. Studies have suggested an association between DM and HNC [13–16]. Tseng et al. [13] found that individuals newly diagnosed with DM had a 1.47-fold higher overall incidence of HNC compared to those without DM, regardless of factors such as age, sex, geographic location, income, and coexisting conditions. A systematic review and meta-analysis study involving more than 51,496 individuals with HNC cancer in East Asia revealed an association between DM and elevated HNC risk [14]. A South Korean national cohort study found that diabetes mellitus (DM) increases the risk of head and neck cancer (HNC) in both genders, with a higher risk compared to moderate alcohol consumption and equal to the risk from cigarette smoking [16]. Therefore, identifying a medication that could mitigate or eliminate the risk of HNC in patients with T2DM is imperative.

H1-antihistamine (AH) use has been linked to a reduction in the risk for various cancers, including glioma, ovarian cancer, and hepatocellular carcinoma [17–19]. One multinational study found that antihistamine (AH) use was linked to a 30% decrease in the risk of adult glioma [17]. AHs were also found to reduce ovarian cancer risk among premenopausal women [18,20]. A retrospective cohort study from 2022 showed that AH use may decrease the risk of hepatocellular carcinoma in a dose-dependent manner among patients infected with hepatitis B virus, hepatitis C virus, or both [19]. Another study found that AH use can lower the risk of HCC in patients with T2DM who are not infected with hepatitis B or C, and the effect is dose-dependent [21]. Despite a growing body of evidence on the anticancer properties of AHs [17–21], the impact of their use on HNC risk in patients with T2DM has not been fully explored. To fill this gap, we performed a nationwide, population-based cohort study using propensity score matching (PSM) to assess the association between AH use and HNC risk in T2DM patients.

## Materials and Methods

### Data sources and study cohort

This study was based on data from the National Health Insurance Research Database (NHIRD) of Taiwan’s National Health Insurance (NHI) system, which covers over 99.6% of the country’s population. The NHIRD provided detailed medical records of NHI enrollees, after anonymizing them to protect patients’ privacy. Information on patient diagnoses, treatments, and drug usage was extracted for analysis. The study was approved by the Institutional Review Board of Tzu-Chi Medical Foundation and conducted following the Strengthening the Reporting of Observational Studies in Epidemiology guidelines and the principles of the Declaration of Helsinki, with a waiver of informed consent obtained.

### Study design and participants

Our T2DM cohort initially consisted of 480,000 patients with at least three ambulatory care claims or one inpatient claim from 2008 to 2020 in the NHIRD. The patients were monitored from their diagnosis until HNC development, death, or cohort exit. The index date was set as the date one year after the start of AH use (at least 28 cumulative defined daily doses [cDDDs]) or cohort entry. The follow-up period lasted one year from the index date. HNC was the endpoint. AH use was defined as using more than 28 cDDDs. In the study, only H1-antihistamines were considered due to their association with reduced risk of cancer, such as glioma, ovarian cancer, and hepatocellular carcinoma in previous studies [17–19]. We did not include H2-antihistamines in our analysis because they have not been shown to have the same protective effect.

We filtered out participants who met any of the following criteria: (1) received a HNC diagnosis in the first year after T2DM diagnosis, (2) missing information on sex and age in the database or below 18 years of age, (3) received a HNC diagnosis in the first year after index date, (4) had a follow-up duration less than 1 year, (5) diagnosed with any type of cancer within 1 year prior to the cohort entry date to eliminate the impact of HNC metastasis.

### AH use

An AH prescription was defined as previously reported in the protocol [19,21]. In this study, AHs were used for various conditions including asthma, allergic rhinitis, medication allergies, environmental allergies, or viral infections (such as itchy eyes, runny nose, and pruritus). Information was collected about the type of drug, dose, method of administration, prescription date, and number of pills dispensed, as AH usage was reimbursed by Taiwan’s National Health Insurance (NHI). To account for different AH use patterns over time, we treated AH usage as a time-varying factor in the Cox model. The cumulative dose was calculated by multiplying the number of pills dispensed by the prescribed dose and dividing the result by the number of days’ supply dispensed. The cDDDs were calculated as the sum of the World Health Organization-defined average daily dose per drug. Occasional use was excluded by defining <28 cDDDs as AH non-use and ≥28 cDDDs as AH use in the cohort.

### PSM and covariates

In our analysis, we used a time-varying Cox proportional hazards model after adjusting for potential confounding factors. This model compared the time from the index date to HNC onset between T2DM patients with and without AH use. To minimize the influence of confounders, we used PSM based on patient variables like age, sex, income, urbanization, antidiabetic medication, comorbidities, smoking, alcohol-related diseases, and diabetes severity. The presence of comorbidities was determined using ICD-9-CM or ICD-10-CM codes based on one inpatient visit or two or more outpatient visits. In addition to these factors, we also considered Epstein-Barr virus and human papillomavirus infection, as well as the indications for H1-antihistamine use (e.g., asthma, dermatitis, rhinitis, and urticaria) [22,23].

In this study, we present continuous variables as means ± standard deviations or medians (first and third quartiles) as appropriate. We utilized PSM with a caliper width of 0.2, [24] using the greedy method to match patients at a 1:1 ratio. Matching is a popular method for selecting control subjects with identical important background covariates, aimed at minimizing differences between patient groups.

### Primary endpoints

The primary end point in this study was the diagnosis of HNC, which was verified through the records found in the Registry for Catastrophic Illness Patients [25].

## Results

### Baseline characteristics of the study population

In total, 480,000 individuals diagnosed with T2DM from 2008 to 2018 were included in the study. The final follow-up date was December 31, 2020. Exclusions were made for patients with HNC within a year of T2DM diagnosis (n = 8,341), those without age or sex data or under 18 years old (n = 12,388), those with HNC within a year of the index date (n = 299), those with less than a year of follow-up (n = 22,370), and those with a history of other cancers (n = 11,454). The remaining participants (N = 425,148) were split into two groups: (1) those who did not use AHs (n = 370,589) and (2) those who did (n = 54,559). After 1:1 matching, each group had 54,384 patients. At baseline, relative to the AH nonusers, the AH users were older, tended to be female, had lower income levels, lived more in rural areas, had more insulin use, were less likely to use antidiabetic medication, had more advanced diabetic severity (higher adapted Diabetes Complications Severity Index scores), had lower rates of cigarette smoking and alcohol-related diseases, had more indications for antihistamine, and had lower CCI scores (Suppl. Table 1). Thus, PSM was employed to establish a proper balance between the cases and control groups (Table 1).

**Table 1:** Characteristics of T2DM patients with and without AH use after propensity score matching

	AH nonusers		AH users		ASMD
	N = 54,384		N = 54,384		
	N	%	N	%	
**Age** (mean ± SD), years	56.88 ± 15.19		56.71 ± 15.08		0.005
Age, median (IQR, Q1, Q3), years	56.00 (45.00,68.00)		56.00 (46.00,69.00)		
Age group, years					0.004
≤50	19,370	35.62	18,613	34.23	
51–60	11,276	20.73	11,016	20.26	
61–70	12,140	22.32	12,203	22.44	
>70	11,598	21.33	12,552	23.08	
**Sex**					0.006
Female	26,661	49.02	26,317	48.39	
Male	27,723	50.98	28,067	51.61	
**Income (NTD)**					0.012
Low income	626	1.15	636	1.17	
10,000–19,999	12,403	22.81	12,340	22.69	
≤20,000	23,344	42.92	23,640	43.47	
20,001–30,000	9,846	18.10	9,678	17.80	
30,001–45,000	5,439	10.00	5,360	9.86	
>45** **000	2,726	5.01	2,730	5.02	
**Urbanization**					0.002
Rural	17,219	31.66	17,323	31.85	
Urban	37,165	68.34	37,061	68.15	
**Antidiabetic medication**					
Insulin	7,511	13.81	10,323	18.98	0.140
Metformin	17,373	31.95	15,995	29.41	0.055
Sulfonylureas	21,875	40.22	18,623	34.24	0.124
Alpha-glucosidase inhibitors	2,196	4.04	2,290	4.21	0.009
Thiazolidinediones	1,700	3.13	1,566	2.88	0.014
Dipeptidyl peptidase-4 inhibitors	2,267	4.17	2,232	4.10	0.003
Sodium–glucose cotransporter-2 inhibitors	253	0.47	196	0.36	0.016
**Antidiabetic medication regimen**					0.036
No antidiabetic medication use	23,257	42.76	23,678	43.54	
One drug	16,219	29.82	16,648	30.61	
Combination of two types	9,676	17.79	9,205	16.93	
Combination of ≥3 types	5,232	9.62	4,853	8.92	
**Diabetes severity**					
**aDCSI Score**					0.075
0	29,537	54.31	28,988	53.30	
1	10,949	20.13	10,158	18.68	
2	8,816	16.21	9,007	16.56	
≥3	5,082	9.34	6,231	11.46	
**aDCSI**					
Retinopathy	3,215	5.91	1,694	3.11	0.028
Nephropathy	5,369	9.87	6,751	12.41	0.025
Neuropathy	4,731	8.70	3,786	6.96	0.017
Cerebrovascular disease	5,527	10.16	6,010	11.05	0.009
Cardiovascular disease	10,948	20.13	14,421	26.52	0.064
Peripheral vascular disease	1,892	3.48	1,789	3.29	0.002
Metabolic disease	1,489	2.74	864	1.59	0.011
**Cigarette smoking**	2,223	4.09	2,068	3.80	0.003
**Alcohol-related diseases**	327	0.60	353	0.65	0.000
**CCI Scores**					
Mean (SD)	0.25 ± 0.76		0.21 ± 0.65		0.055
Median (IQR, Q1–Q3)	0.00 (0.00,0.00)		0.00 (0.00,0.00)		
**CCI Scores**					0.001
0	47,794	87.88	47,838	87.96	
≥1	6,590	12.12	6,546	12.04	
**Comorbidities**					
Congestive heart failure	1,034	1.90	663	1.22	0.007
Dementia	337	0.62	264	0.49	0.001
Chronic pulmonary disease	2,306	4.24	3,624	6.66	0.024
Rheumatic disease	245	0.45	291	0.54	0.001
Liver disease	3,004	5.52	2,064	3.80	0.017
Hemiplegia and paraplegia	272	0.50	330	0.61	0.001
Renal disease	709	1.30	599	1.10	0.002
AIDS	4	0.01	3	0.01	0.000
Hypertension	5,548	10.20	5,535	10.18	0.000
Hyperlipidemia	1,422	2.61	1,425	2.62	0.000
Epstein-Barr virus infection	10,453	19.22	10,273	18.89	0.001
Human papillomavirus infection	9,784	17.99	9,604	17.66	0.001
**Indications for antihistamine**					
Asthma	5,563	10.23	5,433	9.99	0.001
Allergic rhinitis	17,642	32.44	17,093	31.43	0.002
Dermatitis	16,854	30.99	16,620	30.56	0.001
Urticaria	2,833	5.21	2,904	5.34	0.001
					***p***
Mean (+/− SD) follow-up, years	9.26 ± 3.28		12.33 ± 3.73		<0.0001
Median (IQR, Q1, Q3) follow-up, years	9.09 (7.55,10.51)		13.90 (9.64,15.42)		<0.0001
**Head and neck cancer**					0.0040
No	53,836	98.99	54,038	99.36	
Yes	548	1.01	346	0.64	

After conducting PSM, the two groups, AH users and non-users, displayed a balanced age distribution. Furthermore, there were no significant differences between the groups in terms of demographic and clinical factors like age, sex, income, urbanization, antidiabetic medication, antidiabetic medication regimen, comorbidities, smoking habits, alcohol-related diseases, and diabetes severity.

### Risk factors with HNC incidence

Table 2 shows the relationship between HNC risk and co-occurring medications and health issues. Our study found that HNC risk was higher for men than women (HR: 2.35; 95% CI: 1.90 to 5.51). Additionally, smoking cigarettes (HR: 1.21; 95% CI: 1.02 to 1.45) and having alcoholic liver disease (HR: 3.31; 95% CI: 1.78 to 5.70) were linked to a higher HNC risk.

**Table 2:** Predictors of head and neck cancer risk in cox proportional model

	Crude HR	(95% CI)	*p*	aHR*	(95% CI)	*p*
**AH use** (Ref. nonuser)						
AH user	0.59	(0.51, 0.68)	<0.0001	0.55	(0.48, 0.64)	<0.0001
**Sex** (Ref. female)						
Male	5.31	(4.38, 6.44)	<0.0001	2.35	(1.90, 5.51)	<0.0001
**Age group**, years (Ref. 18–50)						
51–60	1.23	(1.03, 1.5)	0.0253	1.24	(0.93, 1.50)	0.0880
61–70	1.17	(0.97, 1.42)	0.0974	1.06	(0.89, 1.30)	0.5099
>70	1.06	(0.86, 1.32)	0.5579	0.90	(0.70, 1.14)	0.4233
**Income** (Ref. Low income, NTD)						
10,000–19,999	0.66	(0.32, 1.36)	0.2611	0.98	(0.50, 2.03)	0.9966
≤20,000	0.97	(0.48, 1.96)	0.9402	1.04	(0.52, 2.07)	0.9334
20,001–30,000	0.87	(0.43, 1.78)	0.7161	0.98	(0.51, 1.99)	0.9773
30,001–45,000	0.89	(0.43, 1.84)	0.7588	0.81	(0.41, 1.70)	0.6103
>45,000	0.63	(0.29, 1.39)	0.2551	0.51	(0.22, 1.12)	0.0891
**Urbanization** (Ref. rural)						
Urban	0.90	(0.78, 1.05)	0.1854	0.90	(0.79, 1.08)	0.2725
**Antidiabetic medications**						
Insulin	1.13	(0.94, 1.35)	0.3892	1.10	(0.91, 1.28)	0.2950
Metformin	0.91	(0.79, 1.21)	0.2643	0.87	(0.84, 1.16)	0.3565
Sulfonylureas	1.20	(0.76, 1.88)	0.2808	1.14	(0.68, 1.72)	0.3408
Alpha-glucosidase inhibitors	1.05	(0.74, 1.25)	0.4902	1.02	(0.74, 1.21)	0.3411
Thiazolidinediones	1.04	(0.71, 1.33)	0.3319	1.04	(0.73, 1.21)	0.2746
Dipeptidyl peptidase-4 inhibitors	1.06	(0.81, 2.85)	0.4254	1.07	(0.78, 2.64)	0.3502
Sodium–glucose cotransporter-2 inhibitors	0.80	(0.43, 2.49)	0.4377	0.77	(0.64, 2.21)	0.3931
**Antidiabetic medication regimen** (Ref. no an antidiabetic use)						
One drug	1.15	(0.96, 1.63)	0.3533	1.07	(0.11, 1.44)	0.2201
Combination of two types	1.19	(0.80, 1.65)	0.3245	1.11	(0.87, 1.50)	0.4514
Combination of ≥3 types	1.26	(0.87, 2.35)	0.4528	1.20	(0.89, 2.13)	0.3404
**Diabetes severity**						
**aDCSI Score** (Ref. 0)						
1	0.99	(0.83, 1.21)	0.9803	0.96	(0.79, 1.17)	0.7733
2	1.20	(0.99, 1.47)	0.0692	1.04	(0.86, 1.28)	0.7269
≥3	1.24	(0.97, 1.59)	0.0834	1.02	(0.80, 1.33)	0.8884
**Cigarette smoking** (Ref. nonsmoker)	1.374	(1.16, 1.63)	0.0003	1.21	(1.02, 1.45)	0.0206
**Alcohol-related diseases** (Ref. no alcohol-related diseases)	4.627	(2.08, 8.46)	0<.0001	3.31	(1.78, 5.70)	<0.0001
**CCI** (Ref. 0)						
CCI ≥ 1	1.491	(1.21, 1.84)	0.0002	1.05	(0.84, 1.36)	0.6043
**Comorbidities**						
Congestive heart failure	1.61	(0.52, 2.35)	0.4729	1.20	(0.57, 2.02)	0.3751
Dementia	0.85	(0.65, 2.51)	0.6928	0.88	(0.64, 2.10)	0.3228
Chronic pulmonary disease	1.28	(0.81, 1.99)	0.2350	1.17	(0.71, 1.57)	0.3212
Rheumatic disease	1.10	(0.56, 2.52)	0.4922	1.09	(0.57, 2.19)	0.5503
Liver disease	1.18	(0.79, 1.81)	0.4245	1.04	(0.65, 1.60)	0.6726
Hemiplegia and paraplegia	2.53	(0.87, 3.08)	0.1492	2.13	(0.74, 2.95)	0.1494
Renal disease	1.41	(0.97, 2.05)	0.0759	1.09	(0.75, 1.59)	0.6170
AIDS	0.95	(0.50, 1.81)	0.7480	1.10	(0.61, 1.98)	0.7377
Hypertension	1.21	(0.88, 1.55)	0.4150	1.21	(0.87, 1.53)	0.2748
Hyperlipidemia	1.29	(0.81, 1.52)	0.3505	1.22	(0.82, 1.46)	0.2965
Epstein-Barr virus infection	1.08	(0.58, 2.54)	0.4704	1.07	(0.55, 2.17)	0.5381
Human papillomavirus infection	1.16	(0.77, 1.79)	0.4023	1.06	(0.63, 1.58)	0.6504
**Indications for antihistamine**						
Asthma	1.39	(0.95, 2.03)	0.1537	1.11	(0.77, 1.61)	0.4358
Allergic rhinitis	1.03	(0.52, 1.77)	0.7278	1.08	(0.59, 1.96)	0.5159
Dermatitis	1.20	(0.86, 1.53)	0.4020	1.19	(0.85, 1.50)	0.2226
Urticaria	1.22	(0.80, 1.51)	0.3449	1.20	(0.80, 1.44)	0.2941

Note: *All covariates in Table 2 are adjusted. Abbreviations: AH, H1-antihistamine; cDDD, cumulative defined daily dose; IQR, interquartile range; NSAID, nonsteroidal anti-inflammatory drug; SD, standard deviation; N, number; aDCSI, adapted Diabetes Complications Severity Index; SMD, standardized mean difference; Ref., reference; AIDS, acquired immunodeficiency syndrome; CCI, Charlson Comorbidity Index; N, number; NTD, New Taiwan dollars.

### Risks of HNC in AH users vs. nonusers: IRRs and aHRs

Table 2 displays the relationship between AH use and HNC risk in the population of patients with T2DM. After adjustments for age, sex, income level, urbanization, antidiabetic medication, antidiabetic medication regimen, comorbidities, cigarette smoking, alcohol-related diseases, and diabetes severity, AH exposure was linked to a reduced risk of HNC (aHR: 0.55; 95% CI, 0.48 to 0.64) and a lower IR (5.16 *vs*. 8.10 per 100,000 person-years) in the T2DM cohort. The IRR (95% CI) was also lower for AH users compared to nonusers (0.63; 0.55 to 0.73) (Table 3).

**Table 3:** IRRs and aHRs for head and neck cancer

	Events	Person-years	IR (per 100,000 person-years)	IRR	95% CI for IRR	*p*
AH use						
Nonuse (≤28 cDDD)	548	503,572.1	8.10	Ref.		
>28	346	670,751.2	5.16	0.63	0.55 to 0.73	<0.0001

Note: *All covariates in Table 2 are adjusted. Abbreviations: AH, H1-antihistamine; cDDD, cumulative defined daily dose; IR, incidence rate; IRR, incidence rate ratio; Ref., reference; CI, confidence interval; cDDD, cumulative defined daily dose; Q, quarter.

**Figure 1:**
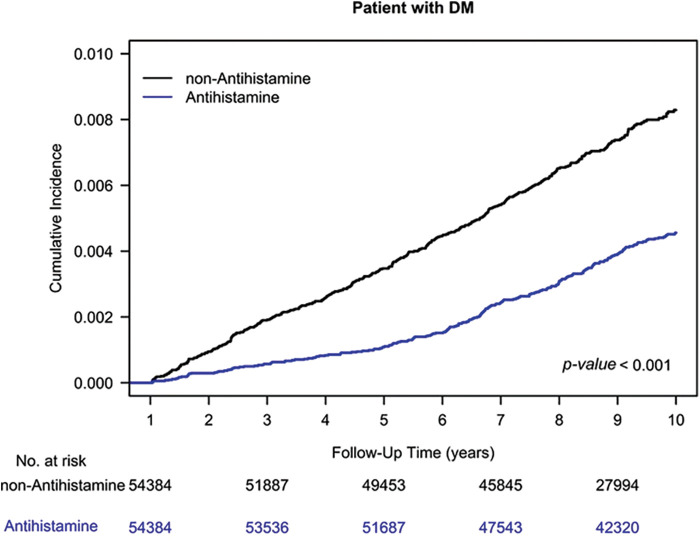
Cumulative incidence of head and neck cancer among AH users and nonusers.

The Kaplan–Meier analysis indicated a reduced HNC risk among AH users compared to nonusers, as shown in Fig. 1 (log-rank test, *p* < 0.001).

## Discussion

The study indicated that individuals with diabetes faced a 1.47 times greater risk of HNC than those without diabetes did for a first malignant tumor (aHR: 1.48; 95% CI, 1.31 to 1.67) [13]. The rise in T2DM cases could result in a similar increase in HNC risk [13,26,27]. As such, finding drugs that could provide protection against HNC among vulnerable groups is crucial. Chronic inflammation and the purging of insulin-like growth factor-1 (IGF-1) caused by T2DM have been linked to HNC risk [20,26]. A preclinical study indicated that AHs could not only relieve allergies, but also exhibit antitumoral properties [28]. This study is a pioneering examination of the benefits of AHs in reducing HNC risk among patients with T2DM. The results, after taking all influencing factors into account, show that HNC risk was lower for those who used AHs compared to those who did not. The IRR for HNC was also found to be lower for AH users compared to non-users.

Previous studies have established various mechanisms that connect T2DM and HNC risk, such as hyperinsulinemia, hyperglycemia, and chronic inflammation [13,26,29–31]. The growth-promoting properties of insulin, as well as its effect on IGF-I, could fuel carcinogenesis, allowing the survival of both preneoplastic and neoplastic cells with insulin, IGF-I, or hybrid receptors [29]. The contribution of elevated IGF-1 receptor expression to HNC drug resistance and poor survival has been documented in previous studies [31]. Hyperglycemia may directly promote tumor growth by sustaining the increased glucose consumption of cancer cells [13,26,29–31]. Additionally, hyperglycemia could initiate cancer development by acetifying DNA damage and oxidative stress [13,26,29–31]. Hyperglycemia-induced chronic inflammation increases HNC risk as well [26]. Hyperglycemia stimulates inflammation, and inflammatory cells secrete cytokines and chemokines that proliferate malignant cells, inhibit apoptosis, aid angiogenesis, and promote metastasis [32]. Therefore, AH use might modulate chronic inflammation and hyperglycemia in those with T2DM, thereby contributing to the reduction of HNC risk [33,34] this conclusion aligns with previous clinical studies [19,21].

The reason behind the protective role of AHs against HNC associated with diabetes is not clear, and several theories have been proposed. The anti-inflammatory effect of AHs can prevent chronic inflammation, which is a risk factor for HNC [33]. Furthermore, AHs stabilize mast cell membranes and block the release of histamine, which has a proliferative effect on malignant cells [34]. Ellegaard et al. [35] reported that only AHs in the cationic amphiphilic drug (CAD) class can reduce cancer mortality. Thus, the anticancer effect of CADs may be related to their CAD structure rather than their antihistamine effect. Therefore, further research is required for elucidating the mechanisms through which AH use reduces diabetes-related HNC risk, apart from the anti-inflammation effects and stabilization of mast cell [33–35].

Conducting a randomized controlled trial (RCT) to assess the impact of AH on HNC risk in T2DM patients is challenging as AH use cannot be altered through tangible interventions [36]. Designing a RCT to evaluate the impact of AHs on HNC risk in patients with T2DM can be difficult due to the challenge of balancing confounding factors between the case and control groups, as well as the need for long-term follow-up in preventive trials [36]. The current study utilized PSM and retrospective real-world data to balance confounding factors between the case and control groups, eliminating potential bias. PSM is considered the gold standard for estimating the impact of covariates when bias may exist. However, PSM cannot control for unmeasured factors and may result in an explicit selection bias, as individuals who cannot be matched are excluded from the inference.

In our study, male sex, cigarette smoking, and alcoholic liver disease were found to be the risk factors for HNC in patients with T2DM, as shown in Table 2. These risk factors align with those reported in previous studies [2–4,37,38]. This result implies that tobacco and alcohol exposures remain the principal HNC risk factors even in a T2DM cohort. The identification of male sex, cigarette smoking, and alcohol consumption as risk factors might be attributed to residual imbalance, [39,40] although well-designed PSM was performed. In our T2DM cohort, all covariates were matched and adjusted; in our T2DM population, AH usage protects against HNC development independently.

This is the first study, to our knowledge, examining the connection between AH use and HNC risk in a T2DM cohort. The study boasts several strengths, such as a large sample size and validation cohort, extended follow-up period, consistent covariates in case and control groups after PSM, and thorough verification of medication records. However, it has some limitations, including its exclusive focus on an Asian population in Taiwan, and therefore, the findings may not be generalizable to non-Asian populations. Nevertheless, the literature does not suggest that ethnicity plays a role in the effect of AHs, their anti-inflammation effects, or their stabilization of mast cells. Second, PSM may not account for unbalanced characteristics and confounders, and thus, any untracked confounding factors could influence the outcome. Third, H1-antihistamines are available over-the-counter (OTC) in Taiwan and are easily reimbursable through national insurance for patients. Patients don’t have to buy H1-antihistamines over-the-counter, leading to a likelihood of higher usage than what our study recorded, given unreported OTC use. If the OTC availability of H1-antihistamines is higher in the non-AH user group, the protective effects of head and neck cancer for AH users may be underestimated. However, our conclusion would not be overturned, only underestimated. Fourth, patients may not have carefully adhered to their AHs regimen, and The estimated AH intake amount may have been too high. However, in the case of nonadherence to the prescribed AH regimen, the effects of AH use on the reduction of HNC risk would be underestimated; our conclusion is thus robust. Fifth, the diagnosis of coexisting conditions in the Taiwan Cancer Registry Database was determined using ICD-9-CM or ICD-10-CM codes from the Taiwan National Health Insurance Research Database. To ensure the accuracy of these diagnoses, the National Health Insurance Administration reviews patients’ medical records and conducts interviews. The Administration also audits hospitals with unusual charges or practices, imposing strict penalties for any malpractice discovered. Despite these efforts, further research in the form of large-scale RCTs comparing the risk of HNC among users and non-users of AH would still be necessary to provide more precise population information, gather disease occurrence data, and minimize confounding variables. However, conducting an RCT in this context may prove challenging.

## Conclusion

Patients with T2DM who use AH have a lower likelihood of HNC, regardless of factors such as age, concurrent health conditions, medications for diabetes, or the severity of their diabetes.

### Statistical analysis

We gathered data on the following patient baseline factors: age (grouped into 10-year increments), sex, income, urbanization, antidiabetic medication type and regimen, coexisting illnesses, smoking, alcohol-related illnesses, diabetes severity, and AH use. The differences between AH users and non-users were analyzed using chi-square and *t*-tests for categorical and continuous variables respectively, with the Wilcoxon rank-sum test for median values. The baseline was established as the date of cohort entry. To determine the HNC risk for each group, we calculated incidence rates and ratios, and adjusted hazard ratios with 95% confidence intervals were estimated using Cox regression models, controlling for baseline characteristics (except for AH use). The cumulative incidence of HNC was estimated using the Kaplan-Meier method and compared through the log-rank test.

The statistical analyses were conducted using SAS for Windows (version 9.4) by the SAS Institute located in Cary, North Carolina, USA. A two-sided *p* value less than 0.05 was deemed statistically significant.

## Data Availability

The data sets supporting the study conclusions are included in the manuscript. We used data from the National Health Insurance Research Database and Taiwan Cancer Registry database. The authors confirm that, for approved reasons, some access restrictions apply to the data underlying the findings. The data used in this study cannot be made available in the manuscript, the supplemental files, or in a public repository due to the Personal Information Protection Act executed by Taiwan’s government, starting in 2012. Requests for data can be sent as a formal proposal to obtain approval from the ethics review committee of the appropriate governmental department in Taiwan. Specifically, links regarding contact info for which data requests may be sent to are as follows: http://nhird.nhri.org.tw/en/Data_Subsets.html#S3 and http://nhis.nhri.org.tw/point.html.

## Abbreviations

aHR: Adjusted hazard ratio
CI: Confidence interval
aDCSI: Adapted Diabetes Complications Severity Index
AH: Antihistamine
cDDD: Cumulative defined daily dose
DDD: Defined daily dose
IQR: Interquartile range
SD: Standard deviation
N: Number
ASMD: Absolute standardized mean difference
HR: Hazard ratio
IR: Incidence rate
IRR: Incidence rate ratio
HNC: Head and neck cancer
T2DM: Type 2 diabetes
PSM: Propensity score matching
NHI: National Health Insurance
NHIRD: National Health Insurance Research Database
IGF-1: Insulin-like growth factor-1
DM: Diabetes mellitus
*ICD-9-CM*: *International Classification of Diseases, Ninth Revision, Clinical Modification*
*ICD-10-CM*: *International Classification of Diseases, Tenth Revision, Clinical Modification*
CAD: Cationic amphiphilic drug
RCT: Randomized controlled trial

## Appendix

**Supplemental Table 1:** Baseline characteristics of AH users and nonusers with T2DM before PSM

	AH Nonusers		AH Users		*p* value
	N = 370,589		N = 54,559		
	N	%	N	%	
**Age** (mean ± SD), years	55.15 ± 14.34		56.77 ± 15.11		<0.0001
Age, median (IQR, Q1, Q3), years	54.00 (45.00,66.00)		57.00 (46.00,69.00)		<0.0001
Age group, years					<0.0001
≤50	131,769	35.56	18,613	34.12	
51–60	99,639	26.89	11,016	20.19	
61–70	73,593	19.86	12,203	22.37	
>70	65,588	17.70	12,727	23.33	
**Sex**					<0.0001
Female	176,025	47.50	26,491	48.55	
Male	194,564	52.50	28,068	51.45	
**Income (NTD)**					<0.0001
Low income	3,774	1.02	637	1.17	
10,000–19,999	89,262	24.09	12,363	22.66	
≤20,000	131,376	35.45	23,786	43.60	
20,001–30,000	75,547	20.39	9,683	17.75	
30,001–45,000	44,453	12.00	5,360	9.82	
>45,000	26,177	7.06	2,730	5.00	
**Urbanization**					<0.0001
Rural	105,921	28.58	17,469	32.02	
Urban	264,668	71.42	37,090	67.98	
**Antidiabetic medication**					
Insulin	46,382	12.52	10,446	19.15	<0.0001
Metformin	126,905	34.24	16,007	29.34	<0.0001
Sulfonylureas	148,024	39.94	18,658	34.20	<0.0001
Alpha-glucosidase inhibitors	17,117	4.62	2,291	4.20	<0.0001
Thiazolidinediones	12,710	3.43	1,566	2.87	<0.0001
Dipeptidyl peptidase-4 inhibitors	16,091	4.34	2,235	4.10	0.0084
Sodium–glucose cotransporter-2 inhibitors	1,771	0.48	196	0.36	0.0001
**Antidiabetic medication regimen**					<0.0001
No antidiabetic medication use	162,541	43.86	23,679	43.40	
One drug	97,129	26.21	16,822	30.83	
Combination of two types	73,233	19.76	9,205	16.87	
Combination of ≥3 types	37,686	10.17	4,853	8.89	
**Diabetes severity**					
**aDCSI Score** (mean ± SD)	0.69 ± 1.12		0.96 ± 1.31		<0.0001
	0.00 (0.00,1.00)		0.00 (0.00,2.00)		<0.0001
**aDCSI Score**					<0.0001
0	232,955	62.86	28,988	53.13	
=1	65,278	17.61	10,158	18.62	
=2	45,713	12.34	9,152	16.77	
≥3	26,643	7.19	6,261	11.48	
**aDCSI**					
Retinopathy	14,088	3.80	1,698	3.11	<0.0001
Nephropathy	33,509	9.04	6,794	12.45	<0.0001
Neuropathy	24,665	6.66	3,793	6.95	0.0097
Cerebrovascular disease	26,999	7.29	6,055	11.10	<0.0001
Cardiovascular disease	68,365	18.45	14,532	26.64	<0.0001
Peripheral vascular disease	9,941	2.68	1,796	3.29	<0.0001
Metabolic disease	6,020	1.62	867	1.59	0.5416
**Cigarette smoking**	54,082	14.59	2,068	3.79	<0.0001
**Alcohol-related diseases**	11,780	3.18	353	0.65	<0.0001
**CCI Scores**					
Mean (SD)	0.80 ± 1.16		0.21 ± 0.65		<0.0001
Median (IQR, Q1–Q3)	0.00 (0.00,2.00)		0.00 (0.00,0.00)		<0.0001
**CCI Scores**					<0.0001
0	225,075	60.73	48,013	88.00	
≥1	145,514	39.27	6,546	12.00	
**Comorbidities**					
Congestive heart failure	17,668	4.77	663	1.22	<0.0001
Dementia	4,288	1.16	264	0.48	<0.0001
Chronic pulmonary disease	47,270	12.76	3,624	6.64	<0.0001
Rheumatic disease	5,929	1.60	291	0.53	<0.0001
Liver disease	78,934	21.30	2,064	3.78	<0.0001
Hemiplegia and paraplegia	5,226	1.41	330	0.60	<0.0001
Renal disease	12,494	3.37	599	1.10	<0.0001
AIDS	104	0.03	3	0.01	0.002
Hypertension	142,254	38.39	5,535	10.14	<0.0001
Hyperlipidemia	70,078	18.91	1,425	2.61	<0.0001
Epstein-Barr virus infection	66,261	17.88	10,311	18.90	0.4024
Human papillomavirus infection	61,295	16.54	9,815	17.66	0.5851
**Indications for antihistamine**					
Asthma	19,752	5.33	5,636	9.99	<0.0001
Allergic rhinitis	65,853	17.77	17,159	31.43	<0.0001
Dermatitis	37,874	10.22	16,679	30.56	<0.0001
Urticaria	9,042	2.44	2,968	5.34	<0.0001
					
Mean (+/- SD) follow-up, years	9.95 ± 3.23		12.32 ± 3.73		<0.0001
Median (IQR, Q1, Q3) follow-up, years	9.92 (7.53,10.26)		13.89 (9.60,15.42)		<0.0001
**Head and neck cancer**					0.008
No	367,851	99.26	54,212	99.36	
Yes	2,738	0.74	347	0.64	

Note: Abbreviations: PSM, propensity scores matching; AH, H1-antihistamine; cDDD, cumulative defined daily dose; IQR, interquartile range; SD, standard deviation; N, number; aDCSI, adapted Diabetes Complications Severity Index; SMD, standardized mean difference; AIDS, acquired immunodeficiency syndrome; CCI, Charlson Comorbidity Index; N, number; NTD, New Taiwan dollars.
